# Diagnosis of adult midgut malrotation in CT: sign of absent retromesenteric duodenum reliable

**DOI:** 10.1186/s13244-025-01921-x

**Published:** 2025-02-17

**Authors:** Min Yang, Shaokun Zheng, Jian Shu, Zhenwei Yao

**Affiliations:** 1 Department of Medical Imaging, Luzhou People’s Hospital, Luzhou, China; 2 Department of Radiology, The Affiliated Hospital of Southwest Medical University, Luzhou, China; 3 Department of Radiology, Huashan Hospital, Fudan University, Shanghai, China

**Keywords:** Midgut malrotation, Adult, Absent retromesenteric duodenum, Computerized tomography

## Abstract

**Objectives:**

To compare the incidence of absent retromesenteric duodenum with other radiological signs and to assess its diagnostic significance for midgut malrotation in adults.

**Methods:**

This IRB-approved retrospective single-center study involved adult patients who underwent abdominal CT scans. Patients were screened for the presence of the absent retromesenteric duodenum sign. Signs observed included the position of the duodenal–jejunal junction (DJJ) and jejunum within the abdomen, the relationship between the superior mesenteric artery (SMA) and superior mesenteric vein (SMV), the locations of the ascending colon, cecum, and appendix, and the presence of intestinal volvulus.

**Results:**

A total of 5594 patients were included. Seven patients exhibited the sign of absent retromesenteric duodenum. Four of these patients were identified as those diagnosed with midgut malrotation in the past five years. The common features observed in all 11 patients were: the horizontal segment of the duodenum did not traverse behind the SMA but instead curved rightwards and forwards adjacent to it; the DJJ and jejunum were positioned in the right abdomen; the SMV was anterior to the SMA. In 7 patients (7/11), the ascending colon, cecum, and appendix were located in the left abdomen. 5 patients (5/11) showed a high cecum position, and 2 patients (2/11) exhibited a pelvic appendix.

**Conclusion:**

The absent retromesenteric duodenum sign in CT diagnosis of adult midgut malrotation has proven to be more reliable.

**Critical relevance statement:**

Radiologists should routinely identify the course of the duodenum horizontal segment in CT images, to prevent misdiagnosis of adult midgut malrotation.

**Key Points:**

- CT is suitable for the diagnosis of adult midgut malrotation.
- Absent retromesenteric duodenum for diagnosing adult midgut malrotation is more reliable than other signs.
- Diagnostic CT criteria for adult midgut malrotation need updating.

**Graphical Abstract:**

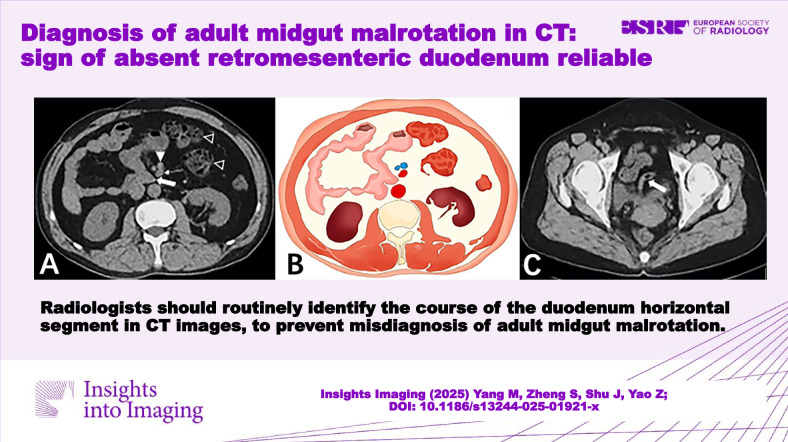

## Introduction

Midgut malrotation is a congenital anomaly that arises during embryonic development when the normal counterclockwise rotation of the midgut, centered around the superior mesenteric artery (SMA), is disrupted, leading to alterations in intestinal positioning [1]. Typically regarded as a pediatric condition, midgut malrotation occurs with an incidence rate of approximately 0.2% between the first month and the first year of life, often leading doctors to overlook its presence in adult patients [2, 3]. Currently, although an increasing number of adult cases of midgut malrotation are accidentally identified through abdominal computerized tomography (CT) scans, the diagnostic criteria and classification remain unclear and inconsistent [4–6].

From an embryological perspective, upon completion of the midgut loop’s 270-degree counterclockwise rotation, the horizontal segment of the duodenum is positioned between the SMA and the aorta, fixed within the retroperitoneal space, marking the end of its embryological journey. Theoretically, in the presence of midgut malrotation, the horizontal segment of the duodenum fails to traverse behind the SMA, remaining instead in the right abdomen. Consequently, Yousefzadeh [7] contends that in the ultrasound diagnosis of midgut malrotation, the criterion of an absent retromesenteric duodenum ought to be employed. However, it remains uncertain whether the absence of the retromesenteric duodenum is more diagnostically valuable for CT scans of adult midgut malrotation than other currently used signs, and whether it will impact the classification of adult midgut malrotation.

The aim of this study is to retrospectively analyze abdominal CT images of adults to compare the incidence of the absent retromesenteric duodenum with other radiological signs and to assess its diagnostic value for adult midgut malrotation.

## Materials and methods

### Patients

This retrospective, single-center study was approved by the Institutional Review Board (approval no. LLW202401008).

The study population consisted of patients who underwent abdominal CT scans at our institution between January and December 2017. Inclusion criteria were age over 18 years, first-time examination during the study period, and undergoing CT plain or enhanced scan of the abdomen. Exclusion criteria were severe image artifacts preventing clear identification of the horizontal segment of the duodenum and a history of duodenectomy. Additionally, patients diagnosed with midgut malrotation in the past five years were retrieved from our institution’s picture archiving and communication system (PACS), and those exhibiting the sign of absent retromesenteric duodenum were observed alongside the study population.

### Imaging acquisition

All patients were scanned using a 32-slice CT scanner (Lightspeed, GE Healthcare), with a scanning range from the diaphragm to the pubic symphysis. The scanning tube voltage was set at 120 KV, and the tube current was automatic. Layer thickness and layer spacing were both 5 mm. Non-emergency patients or those without contraindications drank water or diluted Iohexol 30 min before scanning. Enhanced scans were performed using Iohexol injection at a dose of 1.5–2 mL/kg and an injection rate of 3–4 mL/s. Different scanning phases were selected based on examination requirements, and some patients’ images underwent three-dimensional reconstruction as needed.

### Analysis

Patients were screened for signs of absent retromesenteric duodenum, specifically the horizontal segment of the duodenum not passing behind the SMA. Other signs observed included the position of the duodenal–jejunal junction (DJJ) and jejunum within the abdomen, the relationship between the SMA and superior mesenteric vein (SMV), the positions of the ascending colon, cecum, and appendix, and the presence of intestinal volvulus.

### Statistical analysis

Statistical analysis was conducted using SPSS (version 24.0, IBM Ltd). Normal distribution testing was performed using a Percent Plot. Quantitative data were expressed as mean ± standard deviation, and categorical data were presented as counts with corresponding percentages or fractions.

## Results

A total of 5600 abdominal CT scans were reviewed, with 5 patients excluded due to significant artifacts obscuring the horizontal segment of the duodenum and 1 patient excluded due to a history of duodenectomy. Ultimately, 5594 patients (mean age 58.73 ± 16.68 years, 3097 females) were included. The primary reasons for abdominal CT scans included acute pancreatitis, acute appendicitis, biliary or urinary system stones, cirrhosis, intestinal obstruction, intestinal volvulus, injury, and tumors. Seven patients (mean age 55.86 ± 19.91 years, 3 females, 0.13%) exhibited the sign of absent retromesenteric duodenum. One patient underwent an enhanced scan, while the remaining six underwent only plain scans. One patient had undergone splenectomy, yet the underlying disease remained undiagnosed during the surgical procedure. Notably, only 1 patient (1/7) was correctly identified by the radiologists, avoiding a missed diagnosis. No cases of umbilical hernia, excessive rotation, or reverse rotation were found in the adult patients. Additionally, 4 patients (mean age 54.50 ± 6.14 years, 2 females) diagnosed with midgut malrotation in the past five years were identified.

**Fig. 1:**
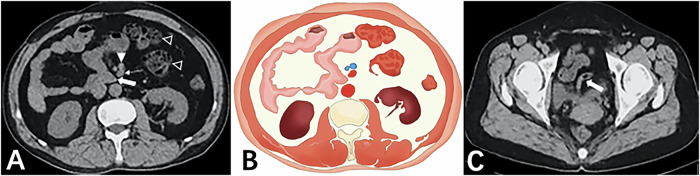
Fifty-two-year-old woman with midgut malrotation. **A** Axial CT plain scan showed the horizontal segment of the duodenum (thick arrow) not traversing behind the SMA (thin arrow), instead curving rightwards and forwards adjacent to the SMA, with the SMV (solid triangle) positioned anterior to the SMA, the jejunum situated in the right abdomen and the ascending colon positioned abnormally in the left abdomen (hollow triangle). **B** Drawing depicted the abnormal midgut malrotation. **C** Axial CT scan showed the pelvic appendix (thick arrow), accompanied by the ascending colon and cecum positioned abnormally in the left abdomen

**Fig. 2:**
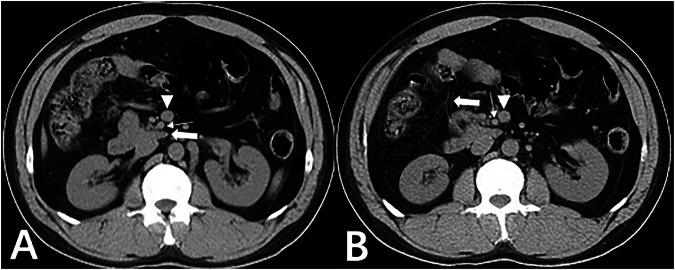
Twenty-two-year-old man with midgut malrotation. **A** Axial CT plain scan showed the horizontal segment of the duodenum (thick arrow) not traversing behind the SMA (thin arrow), instead curving rightwards and forwards adjacent to the SMA, forming a blind end; the SMV (solid triangle) located anterior to SMA. **B** Axial CT scan showed high position and anterior displacement of the cecum and appendix (thick arrow), with the SMV (triangle) rotating clockwise around the SMA (thin arrow)

**Fig. 3:**
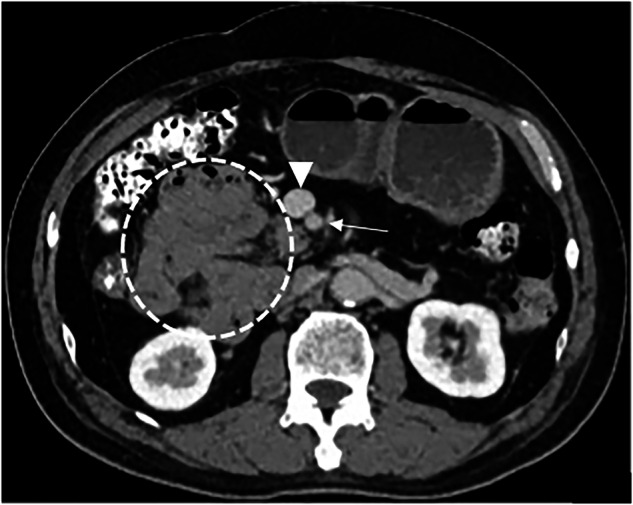
Fifty-nine-year-old man with midgut malrotation. Axial contrast CT showed the jejunum situated in the right abdomen (circle); the SMV (solid triangle) positioned anterior to the SMA (thin arrow)

**Fig. 4:**
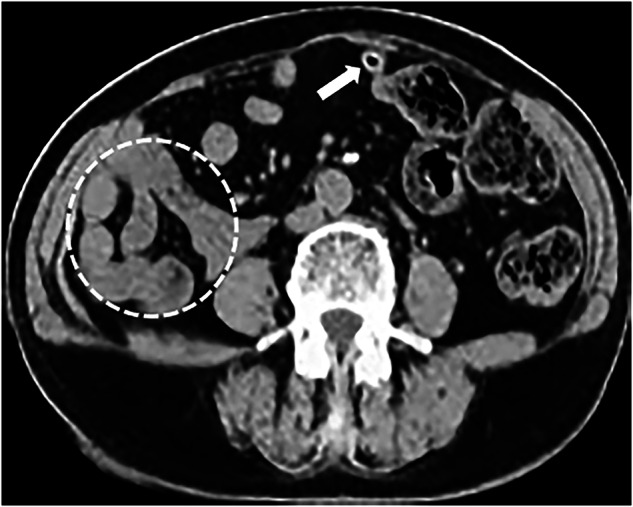
Sixty-year-old woman with midgut malrotation. Axial CT scan showed the jejunum located in the right abdomen (circle), while the ascending colon, cecum, and appendix (thick arrow) located in the left abdomen

The common manifestations among all 11 patients were: (1) the horizontal segment of the duodenum did not traverse behind the SMA but instead curved rightwards and forwards adjacent to it (Figs. 1A and 2A); (2) the DJJ and jejunum were positioned in the right abdomen (Figs. 3 and 4); (3) the SMV was anterior to the SMA (Figs. 1 and 3), and the SMV rotated clockwise around the SMA (Fig. 2). No intestinal volvulus was noted. In 7 patients (7/11), the ascending colon, cecum, and appendix were located in the left abdomen (Fig. 4). Five patients (5/11) exhibited a high cecum position (Fig. 2B). Two patients (2/11) showed a pelvic appendix (Fig. 1C) (Table 1), accompanied by an ascending colon and cecum positioned abnormally in the left abdomen.

**Table 1:** Manifestations of 11 cases

Case	Gender/age	Absent retromesenteric duodenum	DJJ and jejunum in the right abdomen	SMV anterior to SMA	Ascending colon and cecum in the left abdomen	High cecum position	Pelvic appendix
1	M/22	+	+	+	−	+	−
2	M/60	+	+	+	+	+	−
3	M/41	+	+	+	−	+	−
4	F/64	+	+	+	+	+	−
5	M/77	+	+	+	+	−	−
6	F/77	+	+	+	−	−	−
7	F/50	+	+	+	+	−	+
8	F/52	+	+	+	+	−	+
9	F/60	+	+	+	+	−	−
10	M/47	+	+	+	+	+	−
11	M/59	+	+	+	−	−	−
Ratio		100%	100%	100%	63.63%	45.45%	18.18%

*DJJ* duodenal–jejunal junction, *SMV* superior mesenteric vein, *SMA* superior mesenteric artery

**Fig. 5:**
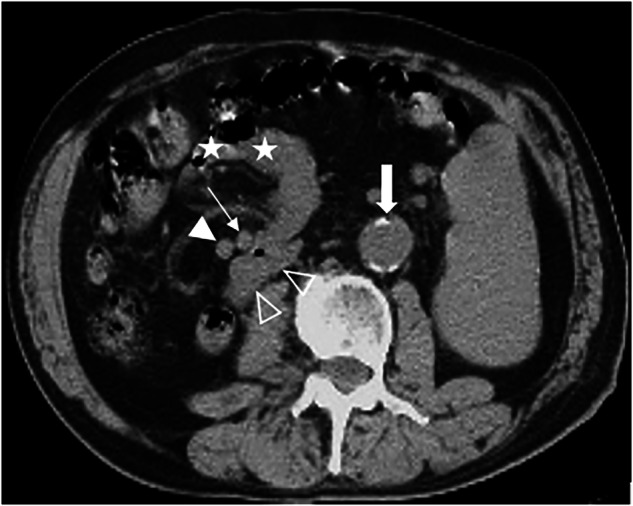
Eighty-one-year-old man with jejunal transposition. Axial CT scan showed the obvious tortuosity and distance between the SMA (thin arrow) and aorta (thick arrow), with the horizontal segment of the duodenum (hollow triangle) traversing normally behind the SMA, the normal relationship of SMA/SMV (solid triangle), the DJJ and jejunum (star) positioned in the right abdomen, forming a reverse “C” shape

**Fig. 6:**
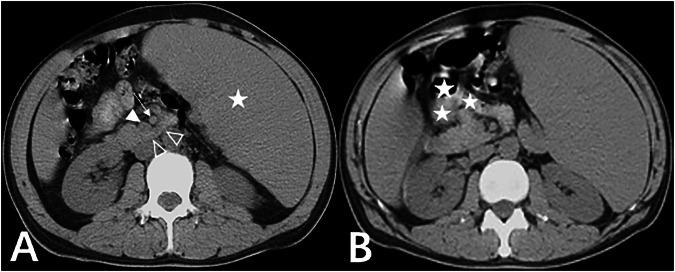
Fourty-five-year-old man with megalosplenia and jejunal transposition. **A** Axial CT scan showed the normal path of the duodenum horizontal segment (hollow triangle) behind the SMA (thin arrow), the normal relationship of the SMA/SMV (solid triangle), the space normally occupied by the jejunum instead occupied by the enlarged spleen (star). **B** Axial CT scan showed the jejunum (star) flipped across the front of mesenteric blood vessels to the right abdomen

Among the 5594 cases, 261 cases (mean age 59.90 ± 17.19 years, 115 females, 4.67%) revealed normal positioning of the duodenum’s horizontal segment behind the SMA, the normal location of DJJ, and the normal relationship between the SMA and SMV, but abnormal jejunum positioning. Due to the partial or complete flipping of the jejunum through the front of the mesenteric blood vessels to the right abdomen, the DJJ, and jejunum presented in a reverse “C” shape (Fig. 5). The positions of the ascending colon, cecum, and appendix were normal, with no intestinal volvulus observed. Among these, 38 patients (38/261) showed that the space normally occupied by the jejunum in the left abdomen was instead occupied by adjacent organs, such as a full stomach, an enlarged spleen (Fig. 6), significantly dilated left renal pelvis and ureter, and an elongated sigmoid colon.

## Discussion

In this study, only seven patients (0.13%) exhibiting the sign of absent retromesenteric duodenum were identified from a cohort of 5594 adult patients. Although the sample size was insufficient to provide an accurate incidence rate estimation, the rarity of this condition aligns with earlier studies conducted in children [8]. Nehra et al [9] found that adults account for 48% of midgut malrotation cases, yet 17% of adult patients remain asymptomatic, potentially leading to a significant underestimation of the number of adults with midgut malrotation. In children, the classic symptoms of bilious vomiting combined with upper gastrointestinal imaging facilitate the diagnosis of midgut malrotation, with intestinal volvulus being the most severe complication [10]. Consequently, some practitioners continue to associate adult midgut malrotation primarily with intestinal volvulus, making it prone to being overlooked in the differential diagnosis of recurrent abdominal pain in adults [11]. It is noteworthy that none of the 11 patients with absent retromesenteric duodenum in this study exhibited intestinal volvulus, suggesting that this may not be a mere coincidence.

Previous diagnostic criteria primarily encompass abnormalities in intestinal morphology and distribution, such as the complete absence of a normal duodenal ring, the jejunum positioned in the right abdomen, most of the colon located in the left abdomen, and the cecum situated in the upper or left abdomen [5, 12]. Whole gastrointestinal X-ray is a well-established method for diagnosing midgut malrotation [9]. However, a whole gastrointestinal X-ray fails to precisely delineate the anatomical relationship between the horizontal segment of the duodenum and the mesenteric vessels.

In this series of abdominal CT scans, the horizontal segment of the duodenum in all 11 patients failed to traverse behind the SMA, instead veering right and advancing adjacent to it. The absence of the retromesenteric duodenum resulted in an abnormal duodenal ring shape and abnormal positioning of the DJJ and jejunum. These signs manifested concurrently, aligning with the X-ray diagnostic criteria for midgut malrotation, thereby reinforcing the prior hypothesis that the absence of a retromesenteric duodenum on CT can confirm this diagnosis.

McVay et al [13] assumed that the abnormal position of the DJJ was a valuable diagnostic indicator for midgut malrotation. This study revealed that in a minority of patients with evident tortuosity of the abdominal aorta, the DJJ and jejunum were situated on the right side of the midline, yet there was a normal course of the duodenal horizontal segment behind the SMA and a normal SMA/SMV relationship (Fig. 5). Although abnormal anatomical relationships of the SMA/SMV have been observed in healthy individuals [14, 15], all 11 patients exhibited this abnormality, with the SMV positioned anterior to the SMA. Consequently, we also endorse the perspective that the normal arrangement of the SMA/SMV disappears, presenting a vertical relationship or left-right inversion, and the clockwise rotation of the SMV around the SMA remains significant in indicating midgut malrotation [5, 10]. Given their potential occurrence in normal individuals, abnormal positions of the DJJ, jejunum, or SMA/SMV observed alone cannot definitively diagnose midgut malrotation.

Previous studies have shown that abnormalities in any stage of intestinal embryonic development can lead to abnormal intestinal position (Table 2), including rotation and fixation abnormalities; rotation anomalies include abnormalities of both cephalic and caudal branches, whereas fixation anomalies predominantly affect the cecum and colon [2, 16]. This study reveals that rotation and fixation anomalies frequently coexist, suggesting that the second and third stages may not be distinct entities. Further research is necessary to confirm this observation. Excessive rotation refers to the total rotation of the midgut exceeding 270 degrees, and reverse rotation denotes an abnormal 90-degree clockwise rotation placing the duodenum on the right side of the abdomen and the transverse colon behind the duodenum [8]. This study did not identify any cases of excessive or reverse rotation. Currently, there is no definitive case report of excessive rotation, and the sole case report of reverse rotation may be a misidentification, as it does not conform to the definition of reverse rotation [5]. Thus, the existence of these two conditions remains questionable.

**Table 2:** Normal and abnormal manifestations at various stages of embryonic development

Developmental stage	Normal	Abnormal
Stage 1	The midgut loop protrudes into the umbilical cavity, resulting in a physiological umbilical hernia, which subsequently rotates 90 degrees counterclockwise within this cavity	Umbilical hernia: the midgut loop fails to retract back into the abdominal cavity
Stage 2	The midgut loop retracts and rotates 180 degrees, positioning the horizontal segment of the duodenum posterior to the SMA, the jejunum in the left abdomen, and the cecum in the right abdomen	Midgut malrotation: the right jejunum and ileum with or without left cecum
		Excessive rotation: the cecum in the right pelvic cavity
		Reverse rotation: anterior ileum
Stage 3	The cecum descends to the lower right quadrant, with the ascending colon and descending colon fixed to the posterior abdominal wall	High position cecum, with or without Ladd’s band: the cecum has not descended to its usual location

*SMA* superior mesenteric artery

In the third stage, abnormal intestinal fixation leads to a high cecum position, observed in five patients in this study and not always accompanied by abnormalities in the second stage. Additionally, two patients exhibited a pelvic appendix with the ascending colon and cecum misplaced in the left abdomen. This anomaly may occur because the transverse colon isn’t fully secured, allowing the appendix to move down into the pelvic area. In cases of appendicitis, such presentations often lead to misdiagnosis, as documented in these literatures [17, 18]. However, when present, this may be a crucial indication of midgut malrotation. An abnormal cecum position may be accompanied by Ladd’s bands or high cecum motility, but these signs are challenging to detect on a single-time CT examination.

McVay et al [13] classified midgut malrotation into classical and atypical forms. In the classical form, the DJJ is located below the pyloric level and to the right of the midline, often accompanied by cecal translocation. In the atypical form, the DJJ is situated below the horizontal line of the pylorus, to the left of the midline, and the jejunum is positioned in the right abdomen. Their study found that adult midgut malrotation was mostly atypical, with mild or absent clinical symptoms [13]. However, if the duodenal horizontal segment runs normally and the DJJ is on the left side of the midline, it suggests that midgut rotation has been completed. Therefore, the “atypical” cases, as seen in our study’s 261 instances, do not conform to the definition of midgut malrotation. In such cases, it is advisable to use the term “jejunal transposition”. In this cohort, the position occupied by the jejunum in 38 jejunal transposition patients was taken by adjacent organs, and all 261 patients had normal mesenteric vascular positions, indicating that jejunal displacement may not be a congenital anomaly but rather a consequence of increased mesenteric activity or space occupation postnatally.

A study by Jayaraman et al [19] revealed that the normal relationship between the SMA and duodenum is observable in the majority of adult abdominal CT scans. In our study, only five out of 5594 patients could not be assessed due to significant artifacts, confirming that the absent retromesenteric duodenum is a readily identifiable sign. We found that both abnormal intestinal distribution and mesenteric blood vessel positioning could be accurately identified on CT plain scan images through continuous layer observation. Enhanced CT scanning and 3D reconstruction provided additional valuable information. While Color Doppler can examine the anatomical structure of the SMA, its diagnostic accuracy (62.5%) is inferior to CT (97.5%) [20]. Therefore, CT scanning is the preferred method for diagnosing midgut malrotation in adults.

Similarly, in our study, one patient underwent splenectomy without a definitive diagnosis during surgery. Only one patient (1/7) was accurately detected by radiologists, highlighting the ease of overlooking this condition if not meticulously examined. If surgeons fail to scrutinize the intestinal structure during operations, they risk overlooking the disease as easily as radiologists do. CT images offer superior visualization of the retroperitoneal duodenum compared to direct inspection. By routinely identifying the anatomical course of the horizontal segment of the duodenum when interpreting abdominal CT images, radiologists can significantly reduce the incidence of missed diagnoses of midgut malrotation.

### Limitations

We acknowledge several limitations. Firstly, it must be emphasized that the sample size is inadequate for accurately estimating the incidence rate of midgut malrotation in adults. Furthermore, it is recognized that there has been no follow-up on confirmed cases to obtain detailed information regarding clinical manifestations and the treatment process. Lastly, given that most patients with this condition do not necessitate surgical intervention, this study was unable to procure data on the sensitivity, specificity, and area under the receiver operating characteristic curve for this diagnostic method.

## Conclusions

In comparison to other indicators, the sign of an absent retromesenteric duodenum in CT diagnosis for adult midgut malrotation has proven to be more reliably indicative. Radiologists should routinely identify the normal course of the duodenum horizontal segment when observing abdominal CT images, to prevent missed diagnoses of midgut malrotation.

## Data Availability

The data from the current study will be available upon reasonable request.

## Abbreviations

CT: Computerized tomography
DJJ: Duodenal–jejunal junction
SMA: Superior mesenteric artery
SMV: Superior mesenteric vein
